# Linking disturbance and resistance to invasion via changes in biodiversity: a conceptual model and an experimental test on rocky reefs

**DOI:** 10.1002/ece3.1956

**Published:** 2016-02-25

**Authors:** Fabio Bulleri, Lisandro Benedetti‐Cecchi, Andrej Jaklin, Ljiljana Iveša

**Affiliations:** ^1^ Dipartimento di BiologiaUniversità di PisaVia Derna 156126PisaItaly; ^2^ Ruđer Bošković InstituteCenter for Marine ResearchG. Paliaga 552210RovinjCroatia

**Keywords:** Biodiversity, biological invasions, Biotic Resistance Hypothesis, canopy‐formers, *Caulerpa cylindracea*, disturbance, habitat‐formers, Intermediate Disturbance Hypothesis, seaweeds

## Abstract

Biological invasions threaten biodiversity worldwide. Nonetheless, a unified theory linking disturbance and resistance to invasion through a mechanistic understanding of the changes caused to biodiversity is elusive. Building on different forms of the disturbance‐biodiversity relationship and on the Biotic Resistance Hypothesis (BRH), we constructed conceptual models showing that, according to the main biodiversity mechanism generating invasion resistance (complementary vs. identity effects), disturbance can either promote or hinder invasion. Following the Intermediate Disturbance Hypothesis (IDH), moderate levels of disturbance (either frequency or intensity) are expected to enhance species richness. This will promote invasion resistance when complementarity is more important than species identity. Negative effects of severe disturbance on invasion resistance, due to reductions in species richness, can be either overcompensated or exacerbated by species identity effects, depending on the life‐traits becoming dominant within the native species pool. Different invasion resistance scenarios are generated when the diversity‐disturbance relationship is negative or positive monotonic. Predictions from these models were experimentally tested on rocky reefs. Macroalgal canopies differing in species richness (1 vs. 2 vs. 3) and identity, were exposed to either a moderate or a severe pulse disturbance. The effects of different canopy‐forming species on the seaweed, *Caulerpa cylindracea*, varied from positive (*Cystoseira crinita*) to neutral (*Cystoseira barbata*) to negative (*Cystoseira compressa*). After 2 years, severely disturbed plots were monopolized by *C. compressa* and supported less *C*. *cylindracea*. Our study shows that the effects of disturbance on invasion depend upon its intensity, the main mechanism through which biodiversity generates invasion resistance and the life‐traits selected within the native species pool. Disturbance can sustain invasion resistance when promoting the dominance of competitively subordinate species possessing traits that allow outperforming invaders.

## Introduction

Concerns over the ecological, social, and economic consequences of the establishment of invasive species have generated a substantial interest in the factors that regulate community resistance to invasion (Mack et al. 2000). An important research thread has focused on the role of native community diversity in driving invasion success (Mack et al. 2000). Although contrasting results have been obtained by experimental versus correlative studies (Fridley et al. 2007), diversity seems to provide a barrier against invasion at small spatial scales (i.e., the Biotic Resistance Hypothesis, BRH; Elton 1958). Greater resistance to invasion at higher levels of native species diversity has been explained by the complementary use of resources among native species (Knops et al. 1999; Naeem et al. 2000; Stachowicz et al. 2002; hereafter referred to as complementary effects) or by a greater probability of including highly competitive species that reduce the amount of resources available to invaders (Huston 1997; Wardle 2001; Arenas et al. 2006; hereafter referred to as species identity effects).

Another research thread has focused on the role of disturbance in determining invasion success (Mack et al. 2000). A positive relationship between disturbance and invasion has been empirically documented in terrestrial and aquatic systems (D'Antonio and Vitousek 1992; Hobbs and Huenneke 1992; Marchetti et al. 2004; Clark and Johnston 2011) and has become one of the most widely accepted truisms in ecology (Lockwood et al. 2013). Natural or anthropogenic disturbance, by decreasing native species richness and/or altering their relative abundances, is predicted to reduce competition with native species and, hence, promote invader establishment (Hobbs and Huenneke 1992; Davis et al. 2000). Recent syntheses (Moles et al. 2012; Jauni et al. 2015) have, however, shown that the relationship between disturbance and invasion can vary from positive to negative, as influenced by a variety of factors, including disturbance and habitat type, or the response variables and temporal scales investigated. As first posited by Daehler (2003) and more recently re‐iterated by Lockwood et al. (2013), one of the main reasons underlying the overgeneralization of positive effects of disturbance on invasion has been the failure to recognize that disturbance frees resources for both native and non‐native species. Facilitation of invasion by disturbance implies non‐native species being characterized by life‐traits that confer them a greater ability than native species to exploit resources becoming available following a disturbance. Regular outperformance of native species by non‐native‐species after a disturbance was not, however, supported by a synthesis of 79 studies on terrestrial plants (Daehler 2003), suggesting that traits selected by disturbance within the native pool do not necessarily imply decreased resistance to invasion.

There has been, however, no formal attempt to merge these two research threads into a unified theory linking disturbance and community resistance to invasion through a mechanistic understanding of the changes caused to biodiversity. Two well‐established, broad ecological concepts may facilitate this process. On the one hand, different forms of the disturbance‐diversity relationship (Connell 1978; Mackey and Currie 2001) provide a conceptual framework to forecast changes in native diversity along a gradient of disturbance intensity. On the other, the BRH provides a conceptual framework for predicting how changes in biodiversity will influence the strength of diversity mechanisms (i.e., complementary and identity effects) that regulate invasion resistance.

Drawing from these two broad ecological concepts, alternative scenarios of invasion resistance can be envisioned according to disturbance levels and to the prevailing mechanism that generates resistance to invasion. The form of the relationship between disturbance (either frequency or intensity) and species richness is still a matter of debate (Mackey and Currie 2001). Predictions of species richness peaking at intermediate levels of disturbance, although holding in several environments (Sousa 1979; Molino and Sabatier 2001; Bongers et al. 2009), cannot be considered as universal (Mackey and Currie 2001), with positive or negative monotonic relationships between disturbance and species richness emerging as reasonably common alternatives (Mackey and Currie 2001).

**Figure 1:**
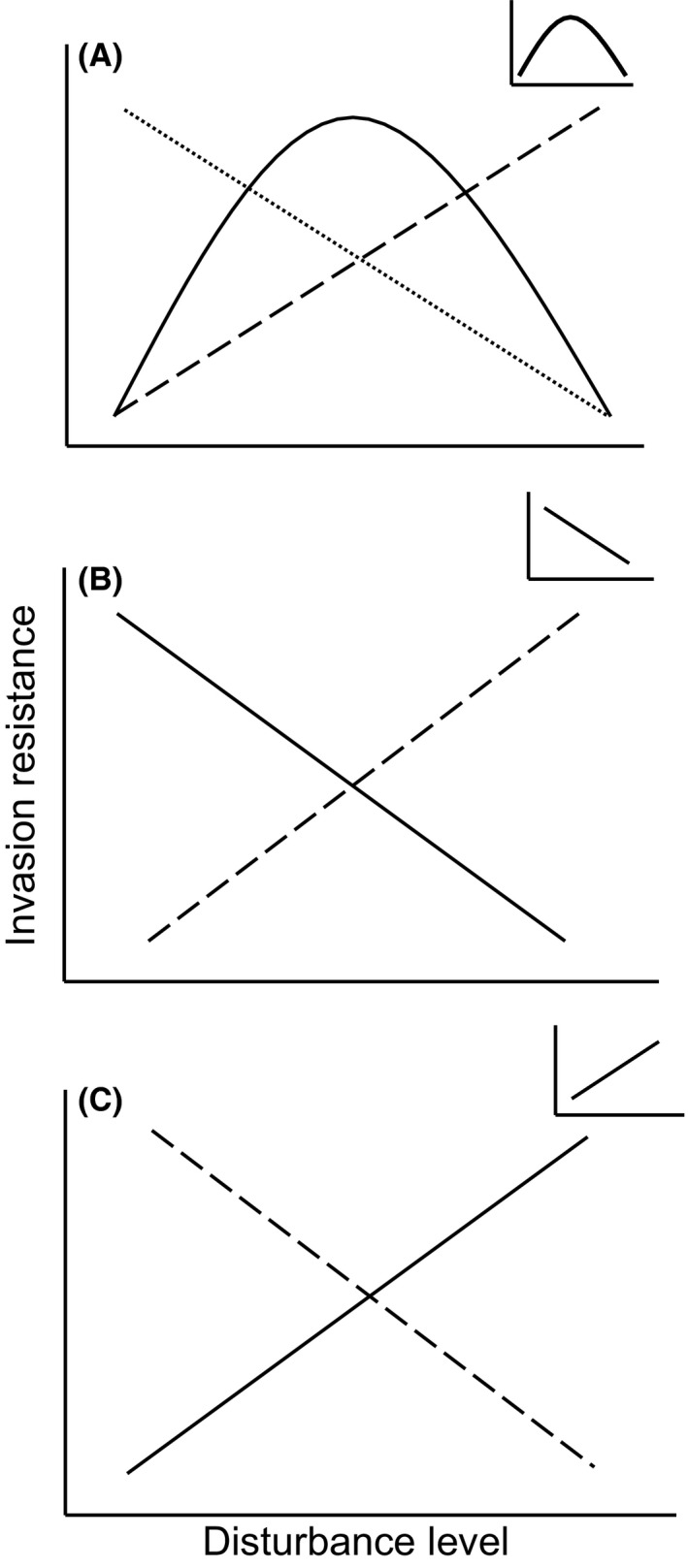
Conceptual models showing how disturbance (frequency or intensity) can influence invasion resistance by altering the relative strength of species complementary and identity effects, under different disturbance‐diversity relationships (each illustrated in the insert, where the x‐axis and the y‐axis represent disturbance levels and species richness, respectively). (A) Following the IDH, species richness is predicted to peak at moderate levels of disturbance, whereas dominance by species characterized by different life‐traits (i.e. competitive vs. opportunistic) is predicted at low and high levels of disturbance. If a strengthening of complementary effects generated by enhanced species richness is associated with decreased relative abundance of poor competitors (i.e., native species not possessing traits that confer them the ability to reduce invader success), invasion resistance will be greater at intermediate levels of disturbance (solid line). High levels of disturbance are predicted to favor species dominance and weaker species complementary effects might be overcompensated by stronger species identity effects if the disturbance promotes dominance of life‐traits that confer invasion resistance (dashed line), or exacerbated when associated with enhanced dominance of life‐traits that do not confer invasion resistance (dotted line). (B) When the disturbance‐diversity relationship is negative monotonic, invasion resistance is expected to decrease with increasing disturbance when a weaker contribution of species complementary effects to invasion resistance is associated with the decline of strong competitors (solid line); by contrast, dominance of strong competitors may sustain invasion resistance at high levels of disturbance (dashed line). (C) When the disturbance‐diversity relationship is positive monotonic, species complementary effects are expected to sustain invasion resistance at high disturbance levels (solid line) when associated to decline of weak competitors; by contrast, dominance of strong competitors may promote invasion resistance at low to moderate levels of disturbance (dashed line).

Following the IDH, intermediate levels of disturbance are expected to enhance local species richness and reduce dominance, potentially strengthening complementary effects (Fig. 1A). The net effect on invasion resistance will be positive when enhanced native species richness is associated with decreased relative abundance of poor competitors (i.e., when complementarity is the main mechanism generating invasion resistance; solid line in Fig. 1A). In contrast, when enhanced native species richness is associated with the decline of relatively strong competitors (e.g., when species identity generates invasion resistance), a strengthening of complementary effects may not be sufficient to offset negative effects due to weakened species identity effects (dashed line in Fig. 1A).

High disturbance levels are predicted to reduce species diversity and promote dominance by disturbance‐tolerant species (Grime 1977; Connell 1978). Although these species are generally weak competitors in relatively benign environments (Grime 1977; Liancourt et al. 2005), they have the potential to regulate the availability of critical resource and to modify abiotic conditions (Bulleri et al. 2009). Negative effects of reducing species richness on invasion resistance (i.e., weakening complementary effects) might be, thus, mitigated or overcompensated by enhanced abundance of species possessing traits able to control invasion (species identity generates invasion resistance; dashed line in Fig. 1A) or exacerbated when reduced native species richness is associated with dominance by poor competitors (dotted line in Fig. 1A).

Different scenarios emerge when the relationship between disturbance and species richness is monotonic. In the case of a negative relationship, invasion resistance would be set to decline with increasing disturbance when decreased strength of complementary effects is associated to the decline of strong competitors (solid line in Fig. 1B). By contrast, disturbance may sustain invasion resistance via species identity effects when promoting the dominance of traits that confer native species the ability to control invasion (dashed line in Fig. 1B). The opposite pattern is expected when the monotonic relationship between disturbance and species richness is positive (Fig. 1C).

Hypotheses from these alternative models were experimentally tested on subtidal rocky reefs in the north‐eastern Mediterranean, supporting both mono‐ and multispecific stands of Fucoids belonging to the genus *Cystoseira* (namely, *C. barbata*,* C. compressa,* and *C. crinita*) and colonized by the exotic green alga, *Caulerpa cylindracea* Sonder (ex *Caulerpa racemosa* (Forsskål) C. Agardh var. *cylindracea* (Sonder) Verlaque, Huisman et Boudouresque; Belton et al. 2014; hereafter referred to as *Caulerpa*). The susceptibility of Fucoids to habitat degradation differs among congeneric species. Along urbanized coasts, the decline of competitively superior species, such as *C. crinita* and *C. barbata*, has promoted the dominance by the more stress/disturbance‐tolerant species, *C. compressa* (Mangialajo et al. 2008; Iveša et al. 2009). In the Mediterranean, invasion by *Caulerpa* has been shown to be hindered by canopy‐forming macrophytes, including Fucoid macroalgae and the seagrass, *Posidonia oceanica* (Bulleri et al. 2010, 2011; Ceccherelli et al. 2014; Tamburello et al. 2014).

Although genetic diversity has been shown to influence ecosystem functioning (Hughes and Stachowicz 2004), the effects of species richness within a genus remain, to the best of our knowledge, unexplored in marine environments. Canopies formed by three *Cystoseira* species are to be considered among the most diverse naturally occurring on Mediterranean rocky reefs. Variations in life‐traits such as the size, toughness, and branching of the thallus and lateral expansion of the holdfast (Appendix S1) among the three *Cystoseira* species could result in different resistance/resilience to disturbance and invasion by *Caulerpa* via either richness or identity effects or both. Previous studies have shown that variations in species or functional group richness levels comparable to those encompassed by our study can influence ecosystem functioning (Duffy et al. 2001; O'Connor and Crowe 2005; Bruno et al. 2008), including resistance to invasion (Arenas et al. 2006). Our study system is, therefore, ideal to test hypotheses regarding potential positive effects of disturbance on invasion resistance via enhanced dominance of competitively subordinate species and/or enhanced species richness.

The effects of a pulse disturbance on the colonization of experimental plots by *Caulerpa* were predicted to vary according to its intensity (i.e., the amount of biomass lost per surface unit; Miller et al. 2011) and to the characteristics (species richness and identity) of extant of canopy‐forming assemblages. Under the Intermediate Disturbance Hypothesis, we expected the application of a disturbance of moderate intensity to increase Fucoids richness and reduce dominance in both mono‐ and two‐species assemblages; this would result in decreased long‐term invasion success of *Caulerpa* if invasion resistance is primarily due to complementary effects or in enhanced success if invasion resistance is regulated by identity effects. Likewise, we predicted that the effects of enhanced species dominance, produced by a severe disturbance, on invasion success could vary from negative to positive, according to the main mechanisms generating resistance and to the traits promoted within the native species pool. We expected severe disturbance to favor *Caulerpa* if the decrease in canopy‐forming species richness was associated with enhanced dominance of weak competitors and to depress it if associated with enhanced dominance of highly suppressive canopy‐forming species.

Finally, when complementarity is the main mechanism underpinning invasion resistance, we expected disturbance to depress resistance from *Caulerpa* invasion when the diversity‐disturbance relationship is negative monotonic and to foster resistance when the relationship is positive monotonic. Alternatively, dominance of competitive traits may promote invasion resistance of canopy stands at high and low levels of disturbance, in the case of a negative or positive monotonic diversity‐disturbance relationship, respectively.

## Materials and Methods

This study was done in the northern Adriatic Sea, about 8 km north of Rovinj (Croatia; 45°8′42″N; 13°35′98″E), from July 2009 to July 2011. At relatively pristine locations along the central Istrian coast, macroalgal assemblages on rocky reefs are dominated by the canopy‐forming species, *Cystoseira barbata*,* C. compressa,* and *C. crinita* (Iveša et al. 2009). At our study site, macroalgal canopies were formed by either one, two, or three species. The invasive macroalga, *Caulerpa cylindracea*, is common on these reefs, where it peaks in abundance (DWg = 1260 ± 221 g m^−2^) in fall/winter (Iveša and Devescovi 2006).

Due to logistic constraints, we did not manipulate the diversity of extant canopy stands, but took advantage of natural variation in their composition. In July 2009, eight 50 × 50 cm quadrats were haphazardly selected, at a depth of 2–3 m, within each of the following types of Fucoid stands (size between 3 and 5 m^2^): (1) mono‐specific stands formed by either *C. barbata* (*bar*.), *C. compressa* (*com*.) or *C. crinita* (*cri*.); (2) 2‐species stands composed by each of the possible pair‐wise combinations (*bar./com*.; *bar./cri*.; *com./cri*.); and (3) 3‐species stands (*bar./com./cri*.). Canopy covers in experimental quadrats, ranging between 80% and 100%, were visually estimated by means of a 50 × 50 cm plastic frame, divided in 25 subquadrats. A score from 0% to 4% was given to each subquadrat (0 when the species is absent; 1 when the species occupies ¾ of the subquadrat; 2 when the species occupies ½ of the subquadrat, etc.) and the percent cover was obtained by summing over the entire set of subquadrats (Dethier et al. 1993). Two‐ and 3‐species quadrats were haphazardly selected at the edges between/among mono‐specific canopy stands in order to maintain species cover even (ratio ~ 1:1). For each type of canopy stand (7 levels), four quadrats were left untouched (controls) and four were randomly assigned to a 50% canopy cover reduction (moderate disturbance). Another four quadrats were randomly selected across the study site, with no regard for the composition in macroalgal canopies, and assigned to the total canopy removal (severe disturbance). The 60 experimental quadrats were scattered over an area ~4000 m^2^, 10s of m apart one from another, and permanently marked at their corners with epoxy putty. Plants and holdfasts, randomly identified within a plot, were removed using a knife. The initial ~1:1 ratio in cover was maintained in species mixtures and, even after the application of the experimental disturbance, quadrats remained entirely embedded within an intact canopy matrix. Levels of disturbance applied simulate realistic effects of episodic, mechanical disturbances, either natural (e.g., large storms; Byrnes et al. 2011) or human (e.g., anchoring and trawling; Bulleri et al. 2011), on stands of canopy‐forming macroalgae. At the beginning of the experiment, *Caulerpa* was removed (if present) from experimental quadrats. The removal of stolons at the inner edge of quadrats often caused their uplifting also from the surrounding area. Thus, in order to standardize the effect of the treatment across plots, *Caulerpa* was removed also throughout a 25‐cm wide external frame. In order to guarantee the complete exclusion of the invader, quadrats were re‐inspected after 2 weeks to remove fragments still present. After 2 years, a lapse of time generally sufficient to allow *Cystoseira* canopy recovery (Bulleri et al. 2002), the cover of *Caulerpa* and that of canopy‐forming species was visually estimated by means of the technique previously described. Macroalgal assemblages, such as those composed by different species of *Cystoseira* that vary in size (Appendix S1), can be multilayered and total cover can exceed 100%.

### Statistical analyses

To test for the effects of disturbance intensity on the relative abundance and richness of canopy‐formers, the cover of each species in total canopy removal plots was first compared with that in the other treatments (0 species vs. Others). The term Others was then partitioned in the terms Canopy type (7 levels) and Disturbance (50% removal vs. Control), treated as fixed, crossed factors. Cochran's tests indicated no departure from the assumption of homogeneity of variances. SNK (Student Newman Keuls) tests were used for *post hoc* comparisons of the means.

A scatter plot suggested a humped shaped relationship between the number of canopy species at the beginning of the experiment and the cover of *Caulerpa*. The effects of initial canopy species richness on the cover of *Caulerpa* were, thus, analyzed by means of polynomial regressions. Although a preliminary ANOVA, conducted following the logic of Beyond BACI designs (Underwood 1991), indicated that differences in *Caulerpa* cover among 1, 2, or 3 species plots varied with disturbance (Among 1–3 species × Disturbance interaction: MS = 1097.837, *F*_6, 55_ = 3.85, *P *<* *0.01), separate regressions were performed on control and disturbed plots to enhance the interpretation of the results. Assumptions of linearity and homogeneity of variances were checked by means of quantile‐quantile and standardized residuals versus fitted values plots. Log transformation of data was effective in improving linearity.

This analysis does not allow formal partitioning of richness (i.e., complementarity) and identity effects at different levels of species richness. Available methods for such task require the determination of the relative contribution of each species to the cumulative effect size in a mixture (Loreau and Hector 2001), which is difficult for many ecosystem functions, such as photosynthesis, nutrient concentration, bioturbation, oxygen flux, and invasion (Ieno et al. 2006). Thus, to get a further insight into the relative role of species richness versus identity effects in multispecies assemblages, we followed the approach of Fargione and Tilman (2005). Since complementarity predicts that diverse assemblages inhibit invasion more than any mono‐culture (Loreau 1998; Stachowicz et al. 2002; Fargione and Tilman 2005), we calculated the percentage of multispecies assemblage plots that had an abundance of the invader lower than that in the most invasion resistant monoculture.

The effects of canopy species identity on *Caulerpa* were formally tested using data from 1 to 2 species assemblages that were analyzed by means of a 3‐way ANOVA including the factors species richness (2 levels, fixed), species composition (3 levels, random and nested within richness) and disturbance (2 levels, fixed and crossed with the other factors). This test does not, however, identify which species, if any, influences the response variable. Thus, following O'Connor and Crowe (2005), a direct test of the effects of the identity of canopy‐forming species was performed by comparing treatments in which one species was present with those in which it was absent. For example, the effects of *C. barbata* were tested through the contrast *bar*., *bar./com*. and *bar./cri* versus *com*., *cri*. and *com./cri*. Since testing for the effects of the different species involved multiple comparisons using the same data, the Bonferroni correction was used to maintain experiment‐wise error rate at 0.05.

## Results

### Response of canopy‐forming assemblages to disturbance

At the end of the study, canopy‐forming species richness was lower in plots where these algae had been totally removed (plots colonized exclusively by *C. compressa*), than in the other treatments (Table S2 and Fig. S2 in Appendix S2). In contrast, there was no effect of a moderate disturbance on canopy‐forming species richness (Table S2 in Appendix S2). Some plots that were mono‐specific at the beginning of the study were colonized by another canopy‐forming species (Fig. S2 in Appendix S2), but the initial ranking of species richness across plots did not change. These results, although not providing conclusive evidence on the form of the diversity‐disturbance relationship, strongly indicate that a severe disturbance caused a decrease in canopy‐forming species richness and promoted dominance, which is consistent with both the Intermediate Disturbance Hypothesis and a negative monotonic diversity‐disturbance relationship.

**Figure 2:**
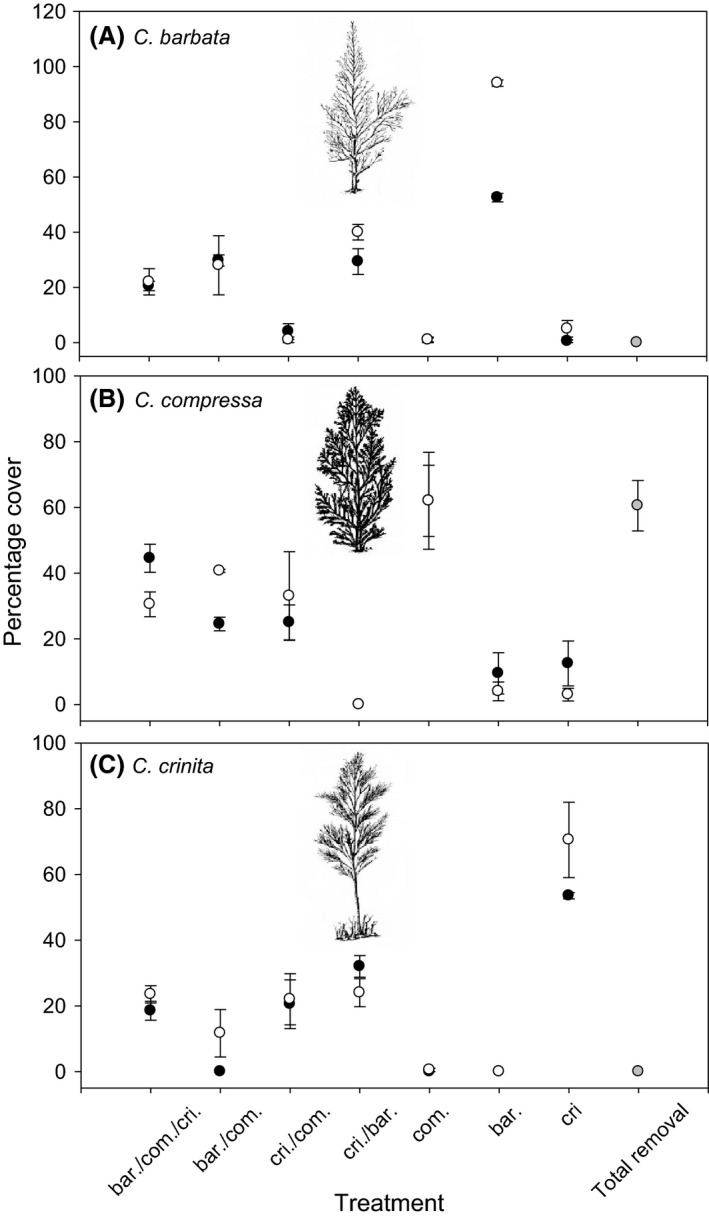
Cover of each of (A) *C. barbata*, (B) *C. compressa* and (C) *C. crinita* at the end of the experiment in experimental plots not disturbed (hollow), exposed to intermediate (black) or severe (gray) disturbance intensity. Data are means + SE;* n *=* *4. Labels on the *x*‐axis refer to the species composition in plots at the beginning of the experiment; the label “total removal” refers to plots exposed to severe disturbance.

Total canopy removal resulted in a significant increment in *C. compressa* cover, in respect to the average value of the other treatments (Fig. 2B; Table S2 in Appendix S2). In contrast, there was no difference in the cover of *C. barbata* or *C. crinita* between total canopy removal plots and the other treatments (Fig. 2B, C). Likely, lack of significant differences was due to large variations in their covers, since neither *C. barbata* nor *C. crinita* colonized total canopy removal plots (Fig. 2). By the end of the experiment, the cover of canopy‐forming species differed among initial canopy types, but there was no effect of disturbance (Fig. 2; Table S2 in Appendix S2). Each of the three species maintained greater covers within their respective monospecific stands than those in which each species was initially present in 2‐ and 3‐species combinations. Although not significant, *C. compressa* cover in stands composed by 3‐species, by *C. barbata* or *C. crinita* alone, was greater in disturbed than undisturbed plots, suggesting that this species was somewhat favored by disturbance.

### Response of *Caulerpa* to experimental treatments

**Figure 3:**
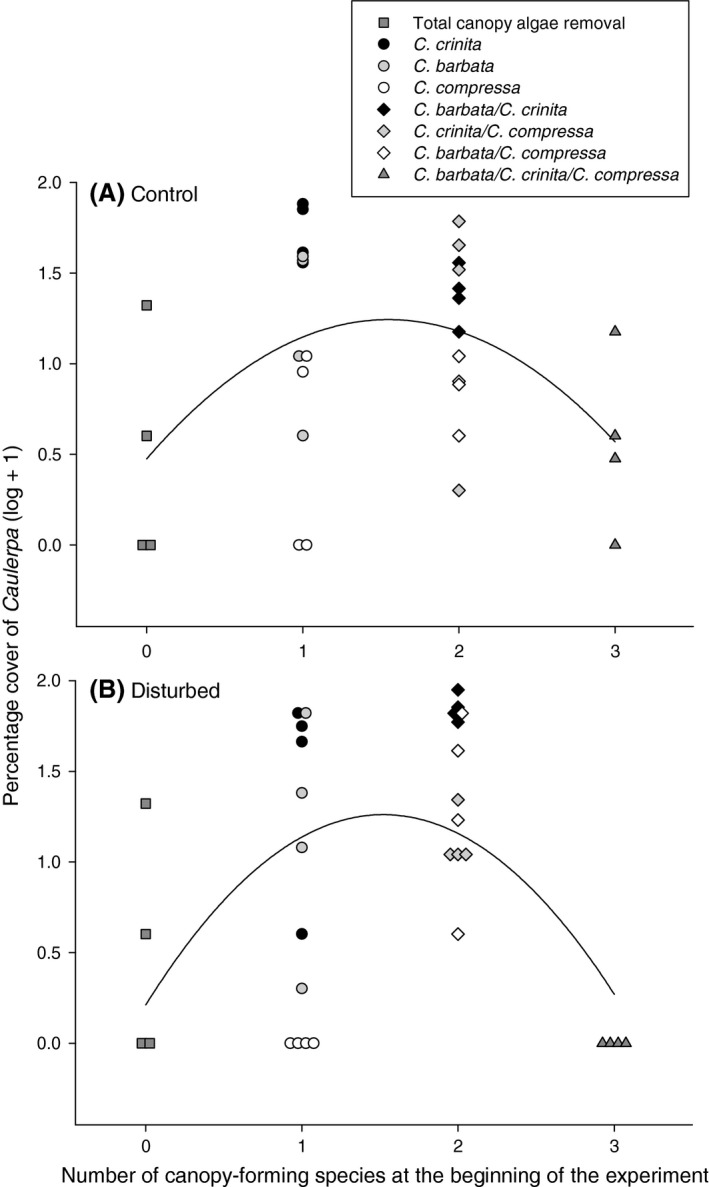
Quadratic regression of the percentage cover of *Caulerpa* against the initial richness of canopy‐forming species in (A) control and (B) disturbed plots. Analysis on log transformed data. Values of *Caulerpa* cover in different 1‐ and 2‐species canopy assemblages are reported with different symbols to enhance the visualization of species identity effects.

There was a significant humped shaped relationship between the number of canopy‐forming species at the beginning of the experiment and the final cover of *Caulerpa* both in control (*y *=* *0.476 + 0.991*x* – 0.320*x* ^*2*^; *P *<* *0.05; linear and quadratic terms: *P *<* *0.01; Fig. 3A) and disturbed plots (*y *=* *0.211 + 1.380*x* – 0.454*x* ^*2*^; *P *<* *0.01; linear and quadratic terms: *P *<* *0.01; Fig. 3B). The cover of *Caulerpa* peaked in 1–2 species plots and was lower in plots that either contained three species or were totally cleared of canopy algae at the beginning of the experiment (0 species). Canopy‐forming species richness explained, however, only ~22% (*R* ^2^ = 0.218) of the variation in *Caulerpa* cover in control plots and ~28% (*R* ^2^ = 0.283) in disturbed plots. Unexplained variation in *Caulerpa* cover was likely due to strong species identity effects within both 1 and 2 species plots; inspection of the graphs suggests that monospecific plots dominated by *C. compressa* had a lower cover of *Caulerpa*, while those dominated by *C. crinita* had a higher invader cover. This pattern was particularly evident in disturbed plots (Fig. 3B). Negative and positive effects of *C. compressa* and *C. crinita*, respectively, on *Caulerpa* were evident also in 2‐species plots (Fig. 3).

In the absence of disturbance, only 17% of 2‐species plots had less *Caulerpa* than the most invasion resistant mono‐culture. In contrast, 75% of the 3‐species plots had less *Caulerpa* cover than the best performing mono‐culture, suggesting that enhanced resistance to invasion was due to complementary effects. Following the application of the disturbance, none of the multispecies assemblages (either 2 or 3 species) had a *Caulerpa* cover lower than that in the most invasion resistant mono‐culture, suggesting no complementary effects.

**Figure 4:**
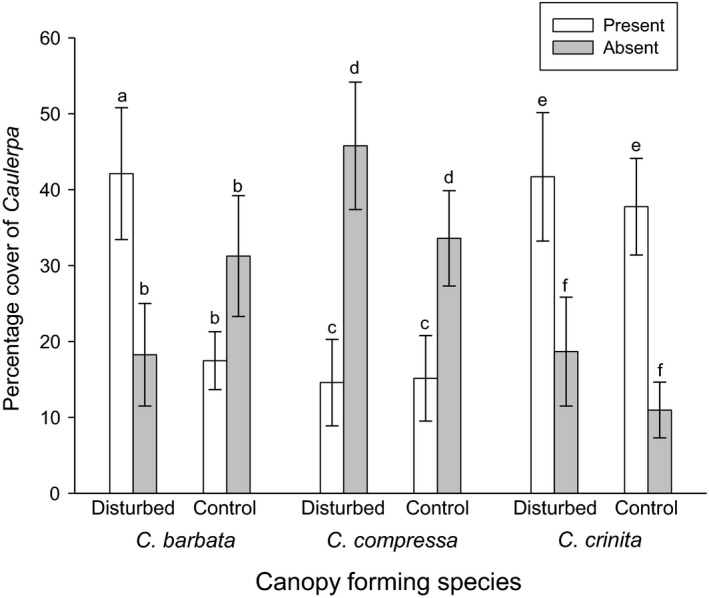
Cover of *Caulerpa* in plots with (hollow bars) or without (gray bars) each of the different canopy‐forming species, separately between disturbed and control plots. Data are means + SE;* n *=* *12. Letters above bars indicate the response of the SNK tests in the case of *C. barbata*; Although SNK tests were not run for *C. compressa* and *C. crinita* (significant main effect of the factor Presence; Table 1), letters are reported above their bars to enhance the visualization of differences.

The ANOVA indicated significant effects of the composition of canopy stands on the cover of *Caulerpa* that varied between control and disturbed plots (significant interaction Composition (Richness) × Disturbance: MS = 1263.738, *F*_4, 36_ = 3.84, *P *<* *0.05; Table S3 in Appendix S3). Planned contrasts revealed that the cover of *Caulerpa* was higher in plots with than without *C. crinita* (Fig. 4; Table 1), whereas the opposite pattern emerged for *C. compressa*. There was no difference in the cover of *Caulerpa* between undisturbed plots with and without *C. barbata*; however, following the disturbance, assemblages with *C. barbata* supported greater covers of the invader than those without (Fig. 4; Table 1).

**Table 1:** Analysis of variance on the effects of the presence of each of the canopy‐forming species and disturbance on the percentage cover of *Caulerpa*

Source of variation		*C. barbata*		*C. compressa*		*C. crinita*	
	df	MS	*F*	MS	*F*	MS	*F*
Presence = P	1	305.021	0.51	7392.058	14.18***	7441.780	14.03***
Disturbance = D	1	406.391	0.68	406.391	0.78	406.391	0.77
P × D	1	4250.058	7.12*	487.687	0.94	42.187	0.08
Residual	44	597.005		521.445		530.440	

*P <* *0.05; ***P <* *0.01.

## Discussion

### Colonization of undisturbed Fucoid stands by *Caulerpa*

Colonization of undisturbed plots by *Caulerpa* was regulated by both the richness and identity of canopy‐forming species. The most diverse plots (3 *Cystoseira* species) and those dominated by *C. compressa* were the least invaded, confirming that species complementary and identity effects can operate simultaneously (Fargione and Tilman 2005).

The effects of macroalgal canopies on *Caulerpa* differed markedly according to the species by which they were formed. The presence of *C. compressa* decreased invasion, that of *C. crinita* enhanced invasion and that of *C. barbata* had no effect. Although we cannot provide definitive evidence of a cause‐effect relationship between the response of *Caulerpa* and the diversity of canopy stands (diversity was not experimentally manipulated), our results strongly suggest that species‐specific attributes of canopy‐formers regulated the colonization of *Caulerpa*. By virtue of the large size of thalli (generally 60 cm, but >2 m in height in some urban areas; L. Iveša pers. obs.), development of large holdfasts and lateral leathery branches in close proximity to the substratum (Appendix S1), *C. compressa* is very efficient in occupying the primary substratum and, likely, in intercepting solar radiation, thereby reducing the establishment and/or persistence of *Caulerpa* (Bernardeau‐Esteller et al. 2015). In contrast, thalli of *C. crinita* are generally smaller (25–30 cm in height), deprived of lateral branches over a large portion of the stipe and characterized by a relatively loose crown of fronds (Appendix S1). A moderate reduction in solar radiation generated by these canopies may have afforded *Caulerpa*, a species well‐adapted to thrive at great depth, in dim light conditions (Piazzi and Balata 2009), a competitive advantage over erect algae and sessile invertebrates (e.g. sponges, Serpulids) that can monopolize space in full and low light conditions, respectively (Bulleri et al. 2002). Finally, *C. barbata* exhibits traits, such as size (up to 50 cm in height) and distance from the substratum of later branches, which are intermediate between *C. compressa* and *C. crinita*. It could be, thus, argued that modification to abiotic conditions and primary space pre‐emption effects by this species were likely negligible in relation to the requirements of the invader.

Worth stressing is that the effects of native Fucoids on *Caulerpa* switched from negative to positive when moving from larger to smaller sized species (i.e., from *C. compressa* to *C. crinita*). Body size is acknowledged among the traits that can regulate the ability of an introduced species to get established into a new region (Davis 2009 and references therein), but little is known about the role it plays in determining resistance to invasion. Our results suggest that the size of resident species, influencing abiotic conditions and resources availability, may represent an important predictor of community resistance to invasion.

Clumping native species into groups on the basis of their functional or morphological attributes is a common approach in biodiversity‐ecosystem functioning experiments (Symstad 2000; Arenas et al. 2006; Bulleri et al. 2009). Opposite effects of different species of *Cystoseira* on space colonization by *Caulerpa* suggest that their clumping into a single group (e.g. canopy‐formers) is not warranted and, more generally, that phylogenetic similarity does not necessarily imply functional similarity. On the other hand, morphological variation within the genus *Cystoseira* promoted resistance to invasion via complementary effects. Although there were only four 3‐species plots, the fact that three of four supported lower *Caulerpa* cover than the most invasion resistant mono‐culture suggests that the co‐occurrence of three macroalgal species differing in shape, size and texture of lateral branches and holdfasts, length of the thallus and width of the canopy crown, reduced the establishment and spread of *Caulerpa*, likely by virtue of a complementary use of resources, such as light and primary space. No such effect emerged in 2‐species plots, indicating that invader outperformance occurred only when extant assemblages include the whole range of traits within the genus *Cystoseira* available at the regional scale.

According to environmental conditions, *Cystoseira* spp. undergo marked variations in morphology (Falace and Bressan 2006) that may influence their effects on invaders. In the Adriatic Sea, *C. compressa* is characterized by a rosette‐shaped form, with flattened primary branches, in fall and winter (Falace and Bressan 2006). Using descriptive data from October 2010, we found a negative correlation between *C. compressa* and *Caulerpa* covers (see Appendix S4), indicating that *C. compressa* limited, to some extent, *Caulerpa* expansion also when characterized by a different thallus morphology. Likewise, the cover of *Caulerpa* was poorly correlated with that of *C. barbata* and positively correlated with that of *C. crinita* (Appendix S4), showing that the direction of *Cystoseira* species effects on the invader, experimentally evaluated during maximum canopy development, persisted when the architectural complexity of their thalli decreased.

### Effects of disturbance on invasion success

A moderate intensity disturbance (50% canopy removal) did not alter the richness of 2‐species canopy stands (mean ± SE: 2.17 ± 0.11). Although it enhanced the mean diversity of mono‐specific assemblages (mean ± SE: 1.75 ± 0.13), it was not sufficient to generate 3‐species stands and, hence, to foster invasion resistance via complementary effects (see above). Species belonging to the genus *Cystoseira*, which are characterized by large and sinking eggs and zygotes, typically have low dispersal (Mangialajo et al. 2007). As shown in other systems, exceeding a disturbance intensity threshold may be necessary to overcome the resilience of extant assemblages (Petraitis and Latham 1999). Likely, resources made available through canopy thinning were not sufficient to allow species other than that (monospecific stands) or those (2‐species stands) already forming the canopy to become established.

Long‐term effects of a moderate intensity disturbance on *Caulerpa* varied according to the initial species composition of canopy stands. Reducing canopy cover of stands which included *C. crinita* or *C. compressa* did not alter their effects on invasion resistance, whereas, after disturbance, stands containing *C. barbata* were more invaded than those lacking this species. Failure of *C. barbata* to recover to reference values in mono‐specific and two‐species (e.g., *cri./bar*.) stands suggests that, after 2 years, a relatively large amount of space was either left unoccupied or monopolized by algal turfs, favoring colonization and/or persistence of *Caulerpa*. Thus, a moderate disturbance applied to *C. barbata* stands, although unable to foster colonization by other canopy‐forming species, was sufficient to free *Caulerpa* from competitive constraints.

Following the application of a moderate disturbance, three‐species and monospecific stands formed by *C. compressa* assemblages were not invaded by *Caulerpa*. This prevents gaining an insight into the relative contribution of complementary and identity effects in generating invasion resistance to invasion at the highest native species richness level. Although previous studies have found no effect of species evenness on invasion (Mattingly et al. 2007), the increase in the cover of *C. compressa* in 3‐species caused by the application of the disturbance may have amplified identity effects without depressing complementarity.

A different scenario emerged when a severe disturbance was applied to native macroalgal stands at the beginning of the experiment. Total removal of macroalgal canopies promoted the formation of monospecific stands of *C. compressa* (see Fig. 4), depressing colonization by *Caulerpa*. Thus, the intensity of disturbance played a pivotal role in regulating community invasion resistance by determining the size of the niche opportunity and, hence, the intensity of the positive or negative effects delivered to native or exotic opportunistic species. Spatial scale and intensity of disturbance are key in determining the magnitude of the competitive release afforded to opportunistic species (Norkko et al. 2006). The formation of mono‐specific stands of *C. compressa* following total canopy clearing is in accordance with predictions, from either a peaked (IDH) or a negative monotonic diversity‐disturbance relationship, of low diversity and dominance of disturbance‐adapted species at high levels of disturbance (Grime 1977; Connell 1978; Mackey and Currie 2001). Although we could not unambiguously distinguish between these two forms of the diversity‐disturbance relationship, resistance to invasion in our study system would be best described by the dashed lines in Figure 1A and B.

Our results do not rule out the possibility that cleared plots might have been colonized by *Caulerpa* at some stage after the disturbance was applied. Reproduction through fragments and rapid elongation of prostrate stolons allow rapid colonization of surfaces by this species (Ceccherelli and Piazzi 2001; Bulleri and Benedetti‐Cecchi 2008; Bulleri et al. 2008). Nonetheless, *C. compressa* was able to either prevent the colonization or persistence of *Caulerpa* in total canopy removal plots. Thus, the relationship between disturbance and resistance to invasion can be either positive or negative, according to the life‐history traits selected within the native species pool. As suggested in Figure 1, positive effects of intense disturbance on invasion resistance can be expected whenever weakened species richness effects are overcompensated by the selection of native traits that confer competitive advantage over potential invaders.

Positive effects of dominance by highly competitive species on community resistance to invasion from *Caulerpa* have been recorded also at low levels of disturbance. On sandy bottoms, mono‐specific meadows formed by the seagrass, *Posidonia oceanica*, have been shown to become susceptible to invasion by *Caulerpa* only when their integrity is disrupted by either natural (e.g., grazing) or human (e.g., anchoring, trawling) disturbances (Ceccherelli et al. 2014; Tamburello et al. 2014). This suggests that dominance by species characterized by marked differences in competitive ability (Ceccherelli et al. 2014; Tamburello et al. 2014) and tolerance to disturbance (this study) may confer resistance to invasion at both ends of a disturbance gradient, as long as, by virtue of their life‐traits, they generate adverse conditions for the invader.

## Conclusions

In summary, our results suggest that the interaction between disturbance intensity and life‐histories of both native and exotic species can determine invasion success. According to the mechanism/s through which native biodiversity generates resistance to invasion (i.e., species complementary and identity effects), the relationship between disturbance intensity and resistance to invasion can assume different shapes. In the case in which a single diversity mechanism is overarching, resistance to invasion can be expected to peak at different points along a gradient of disturbance intensity (i.e., at either low, intermediate or high intensity of disturbance; Fig. 1). Our experiment, documenting negative effects on invasion success of both canopy‐forming species richness and dominance by a competitively subordinated species, brings some support to these conceptual models. Competitive and ruderal strategies are expected to be dominant when disturbance intensity is low and high respectively (Grime 1977). Knowledge of life traits associated to these strategies (Grime 1977), as well as those that characterize a given invader, may form the basis for predicting how dominance, at either low or high intensity of disturbance, may influence invasion success.

Enhanced resistance to invasion following an intense disturbance is not at odds with widely documented positive effects of disturbance on invasibility (Daehler 2003; Moles et al. 2012; Lockwood et al. 2013; Jauni et al. 2015) but rather calls for a shift from a neutral‐ to a niche‐based analysis of the disturbance‐invasion relationship. Framing research within ecological niche theory has advanced our understanding of the mechanisms regulating invasion success (Shea and Chesson 2002) and life‐history traits of both invaders and native communities are recognized as central drivers of invasion success (Kolar and Lodge 2002; Bulleri et al. 2008). This tight link between species life‐traits and niche opportunities seems, however, loosely endorsed within the context of the disturbance‐invasion relationship. Our conceptual model, drawing from broad ecological theories, such as the IDH and the BRH, may provide a useful framework to predict the effects of disturbance on invasion success through a mechanistic understanding of underlying processes. Experimental studies encompassing broader gradients of disturbance and native species richness and testing for the effects of more attributes of disturbance (e.g. frequency, extent, and timing) will be, however, crucial to establish the link between disturbance and invasion via alterations in native diversity.

## Conflict of Interest

None declared.

## Supporting information


**Appendix S1.** Description of thallus morphology of the different species of *Cystoseira*.

**Appendix S2.** ANOVAs comparing the cover of the different canopy‐forming macroalgae and species richness among treatments.

**Appendix S3.** ANOVA assessing the effects of disturbance, canopy species richness and composition on *Caulerpa*.

**Appendix S4.** Relationships between *Caulerpa* and *Cystoseira* spp. cover in fall 2010.

## References

[ece31956-bib-0001] Arenas, F. , I. Sanchez , S. J. Hawkins , and S. R. Jenkins . 2006 The invasibility of marine algal assemblages: role of functional diversity and identity. Ecology 87:2851–2861. 17168029 10.1890/0012-9658(2006)87[2851:tiomaa]2.0.co;2

[ece31956-bib-0002] Belton, G. S. , W. F. Prud'homme van Reine , J. M. Huisman , S. G. A. Draisma , and C. F. D. Gurgel . 2014 Resolving phenotypic plasticity and species designation in the morphologically challenging *Caulerpa racemosa*‐*peltata* complex (Caulerpaceae, Chlorophyta). J. Phycol. 50:32–54. 26988007 10.1111/jpy.12132

[ece31956-bib-0003] Bernardeau‐Esteller, J. , J. M. Ruiz , F. Tomas , J. M. Sandoval‐Gil , and L. Marín‐Guirao . 2015 Photoacclimation of *Caulerpa cylindracea*: light as a limiting factor in the invasion of native Mediterranean seagrass meadows. J. Exp. Mar. Biol. Ecol. 465:130–141.

[ece31956-bib-0004] Bongers, F. , L. Poorter , W. D. Hawthorne , and D. Sheil . 2009 The intermediate disturbance hypothesis applies to tropical forests, but disturbance contributes little to tree diversity. Ecol. Lett. 12:798–805. 19473218 10.1111/j.1461-0248.2009.01329.x

[ece31956-bib-0005] Bruno, J. F. , K. E. Boyer , J. E. Duffy , and S. C. Lee . 2008 Relative and interactive effects of plant and grazer richness in a benthic marine community. Ecology 89:2518–2528. 18831173 10.1890/07-1345.1

[ece31956-bib-0006] Bulleri, F. , and L. Benedetti‐Cecchi . 2008 Facilitation of the introduced green alga *Caulerpa racemosa* by resident algal turfs: experimental evaluation of underlying mechanisms. Mar. Ecol. Prog. Ser. 364:77–86.

[ece31956-bib-0007] Bulleri, F. , L. Benedetti‐Cecchi , S. Acunto , F. Cinelli , and S. J. Hawkins . 2002 The influence of canopy algae on vertical patterns of distribution of low‐shore assemblages on rocky coasts in the northwest Mediterranean. J. Exp. Mar. Biol. Ecol. 267:89–106.

[ece31956-bib-0008] Bulleri, F. , J. F. Bruno , and L. Benedetti‐Cecchi . 2008 Beyond competition: incorporating positive interactions between species to predict ecosystem invasibility. PLoS Biol. 6:e162. 18578573 10.1371/journal.pbio.0060162 PMC2435158

[ece31956-bib-0009] Bulleri, F. , L. Tamburello , and L. Benedetti‐Cecchi . 2009 Loss of consumers alters the effects of resident assemblages on the local spread of an introduced macroalga. Oikos 118:269–279.

[ece31956-bib-0010] Bulleri, F. , D. Balata , I. Bertocci , L. Tamburello , and L. Benedetti‐Cecchi . 2010 The seaweed *Caulerpa racemosa* on Mediterranean rocky reefs: from passenger to driver of ecological change. Ecology 91:2205–2212. 20836441 10.1890/09-1857.1

[ece31956-bib-0011] Bulleri, F. , T. Alestra , G. Ceccherelli , L. Tamburello , S. Pinna , N. Sechi , et al. 2011 Determinants of *Caulerpa racemosa* distribution in the north‐western Mediterranean. Mar. Ecol. Prog. Ser. 431:55–67.

[ece31956-bib-0012] Byrnes, J. E. , D. C. Reed , B. J. Cardinale , K. C. Cavanaugh , S. J. Holbrooks , and R. J. Schmitts . 2011 Climate‐driven increases in storm frequency simplify kelp forest food webs. Glob. Change Biol. 17:2513–2524.

[ece31956-bib-0300] Ceccherelli, G. , and L. Piazzi . 2001 Dispersal of Caulerpa racemosa fragments in the Mediterranean: lack of detachment time effect on establishment. Bot. Mar. 44:209–213.

[ece31956-bib-0013] Ceccherelli, G. , S. Pinna , V. Cusseddu , and F. Bulleri . 2014 The role of disturbance in promoting the spread of the invasive seaweed *Caulerpa racemosa* in seagrass meadows. Biol. Invasions 16:2737–2745.

[ece31956-bib-0014] Clark, G. F. , and E. L. Johnston . 2011 Temporal change in the diversity‐invasibility relationship in the presence of a disturbance regime. Ecol. Lett. 14:52–57. 21070561 10.1111/j.1461-0248.2010.01550.x

[ece31956-bib-0015] Connell, J. H. 1978 Diversity in tropical rainforests and coral reefs – high diversity of trees and corals is maintained only in a non‐equilibrium state. Science 199:1302–1310. 17840770 10.1126/science.199.4335.1302

[ece31956-bib-0016] Daehler, C. C. 2003 Performance comparisons of co‐occurring native and alien plants: implications for conservation and restoration. Annu. Rev. Ecol. Evol. Syst. 34:183–211.

[ece31956-bib-0017] D'Antonio, C. M. , and P. M. Vitousek . 1992 Biological invasions by exotic grasses, the grass fire cycle, and global change. Annu. Rev. Ecol. Evol. Syst. 23:63–87.

[ece31956-bib-0018] Davis, M. A. 2009 Invasion biology. Oxford Univ. Press, New York.

[ece31956-bib-0019] Davis, M. A. , J. P. Grime , and K. Thompson . 2000 Fluctuating resources in plant communities: a general theory of invasibility. J. Ecol. 88:528–534.

[ece31956-bib-0020] Dethier, M. N. , E. S. Graham , S. Cohen , and L. M. Tear . 1993 Visual versus random‐point percent cover estimations: ‘objective’ is not always better. Mar. Ecol. Prog. Ser. 110:9–18.

[ece31956-bib-0021] Duffy, J. E. , K. S. MacDonald , J. M. Rhode , and J. D. Parker . 2001 Grazers diversity, functional redundancy, and productivity in sea grass beds: an experimental test. Ecology 82:2417–2434.

[ece31956-bib-0022] Elton, C. S. 1958 The ecology of invasions by animals and plants. Methuen, London.

[ece31956-bib-0023] Falace, A. , and G. Bressan . 2006 Seasonal variations of *Cystoseira barbata* (Stackhouse) C. Agardh frond architecture. Hydrobiologia 555:193–206.

[ece31956-bib-0024] Fargione, J. E. , and D. Tilman . 2005 Diversity decreases invasion via both sampling and complementarity effects. Ecol. Lett. 8:604–611.

[ece31956-bib-0025] Fridley, J. D. , J. J. Stachowicz , S. Naeem , D. F. Sax , E. W. Seabloom , M. D. Smith , et al. 2007 The invasion paradox: reconciling pattern and process in species invasions. Ecology 88:3–17. 17489447 10.1890/0012-9658(2007)88[3:tiprpa]2.0.co;2

[ece31956-bib-0026] Grime, J. P. 1977 Evidence for existence of 3 primary strategies in plants and its relevance to ecological and evolutionary theory. Am. Nat. 111:1169–1194.

[ece31956-bib-0027] Hobbs, R. J. , and L. F. Huenneke . 1992 Disturbance, diversity, and invasion: implications for conservation. Conserv. Biol. 6:324–337.

[ece31956-bib-0028] Hughes, A. R. , and J. J. Stachowicz . 2004 Genetic diversity enhances the resistance of a seagrass ecosystem to disturbance. Proc. Natl Acad. Sci. USA 101:8998–9002. 15184681 10.1073/pnas.0402642101 PMC428461

[ece31956-bib-0029] Huston, M. A. 1997 Hidden treatments in ecological experiments: re‐evaluating the ecosystem function of biodiversity. Oecologia 110:449–460. 10.1007/s004420050180 28307235

[ece31956-bib-0030] Ieno, E. N. , M. Solan , P. Batty , and G. Pierce . 2006 How biodiversity affects ecosystem functioning: roles of infaunal species richness, identity and density in the marine benthos. Mar. Ecol. Prog. Ser. 311:263–271.

[ece31956-bib-0031] Iveša, L. , and M. Devescovi . 2006 Seasonal vegetation patterns of the introduced *Caulerpa racemosa* (Caulerpales, Chlorophyta) in the northern Adriatic Sea (Vrsar, Croatia). Period. Biol. 108:111–116.

[ece31956-bib-0032] Iveša, L. , D. M. Lyons , and M. Devescovi . 2009 Assessment of the ecological status of north‐eastern Adriatic coastal waters (Istria, Croatia) using macroalgal assemblages for the European Union Water Framework Directive. Aquat. Conserv. 19:14–23.

[ece31956-bib-0033] Jauni, M. , S. Gripenberg , and S. Ramula . 2015 Non‐native plant species benefit from disturbance: a meta‐analysis. Oikos 124:122–129.

[ece31956-bib-0034] Knops, J. M. H. , D. Tilman , N. M. Haddad , S. Naeem , C. E. Mitchell , J. Haarstad , et al. 1999 Effects of plant species richness on invasion dynamics, disease outbreaks, insect abundances and diversity. Ecol. Lett. 2:286–293. 10.1046/j.1461-0248.1999.00083.x 33810630

[ece31956-bib-0035] Kolar, C. S. , and D. M. Lodge . 2002 Ecological predictions and risk assessment for alien fishes in North America. Science 298:1233–1236. 12424378 10.1126/science.1075753

[ece31956-bib-0036] Liancourt, P. , R. M. Callaway , and R. Michalet . 2005 Stress tolerance and competitive‐response ability determine the outcome of biotic interactions. Ecology 86:1611–1618.

[ece31956-bib-0037] Lockwood, J. L. , M. F. Hoopes , and M. P. Marchetti . 2013 Invasion ecology, 2nd ed Blackwell, Oxford.

[ece31956-bib-0038] Loreau, M. 1998 Biodiversity and ecosystem function: a mechanistic model. Proc. Natl Acad. Sci. USA 95:5632–5636. 9576935 10.1073/pnas.95.10.5632 PMC20430

[ece31956-bib-0039] Loreau, M. , and A. Hector . 2001 Partitioning selection and complementarity in biodiversity experiments. Nature 412:72–76. 11452308 10.1038/35083573

[ece31956-bib-0040] Mack, R. N. , D. Simberloff , W. M. Lonsdale , H. Evans , M. Clout , and F. A. Bazzaz . 2000 Biotic invasions: causes, epidemiology, global consequences, and control. Ecol. Appl. 10:689–710.

[ece31956-bib-0041] Mackey, R. L. , and D. J. Currie . 2001 The diversity‐disturbance relationship: is it generally strong and peaked? Ecology 82:3479–3492.

[ece31956-bib-0042] Mangialajo, L. , M. Chiantore , M.‐L. Susini , A. Meinesz , R. Cattaneo‐Vietti , and T. Thibaut . 2007 Zonation patterns and interspecific relationships of fucoids in microtidal environments. J. Exp. Mar. Biol. Ecol. 412:72–80.

[ece31956-bib-0043] Mangialajo, L. , M. Chiantore , and R. Cattaneo‐Vietti . 2008 Loss of fucoid algae along a gradient of urbanisation, and structure of benthic assemblages. Mar. Ecol. Prog. Ser. 358:63–74.

[ece31956-bib-0044] Marchetti, M. P. , T. Light , P. B. Moyle , and J. Viers . 2004 Fish invasions in California watersheds: characteristics of successful and failed invaders. Ecol. Appl. 14:587–596.

[ece31956-bib-0045] Mattingly, W. B. , R. Hewlate , and H. L. Reynolds . 2007 Species evenness and invasion resistance of experimental grassland communities. Oikos 116:1164–1170.

[ece31956-bib-0046] Miller, A. D. , S. H. Roxburgh , and K. Shea . 2011 How frequency and intensity shape diversity‐disturbance relationships. Proc. Natl Acad. Sci. USA 108:5643–5648. 21422284 10.1073/pnas.1018594108 PMC3078405

[ece31956-bib-0047] Moles, A. T. , H. Flores‐Moreno , S. P. Bonser , D. I. Warton , A. Helm , L. Warma , et al. 2012 Invasions: the trail behind, the path ahead, and a test of a disturbing idea. J. Ecol. 100:116–127.

[ece31956-bib-0048] Molino, J.‐F. , and D. Sabatier . 2001 Tree diversity in tropical rain forests: a validation of the intermediate disturbance hypothesis. Science 294:1702–1704. 11721052 10.1126/science.1060284

[ece31956-bib-0049] Naeem, S. , J. M. H. Knops , D. Tilman , K. M. Howe , T. Kennedy , and S. Gale . 2000 Plant diversity increases resistance to invasion in the absence of covarying extrinsic factors. Oikos 91:97–108.

[ece31956-bib-0050] Norkko, A. , R. Rosenberg , S. F. Thrush , and R. B. Whitlatch . 2006 Scale‐ and intensity‐dependent disturbance determines the magnitude of opportunistic response. J. Exp. Mar. Biol. Ecol. 330:195–207.

[ece31956-bib-0051] O'Connor, N. E. , and T. P. Crowe . 2005 Biodiversity loss and ecosystem functioning: distinguishing between number and identity of species. Ecology 86:1783–1796.

[ece31956-bib-0052] Petraitis, P. S. , and R. E. Latham . 1999 The importance of scale in testing the origins of alternative community states. Ecology 80:429–442.

[ece31956-bib-0053] Piazzi, L. , and D. Balata . 2009 Invasion of alien macroalgae in different Mediterranean habitats. Biol. Invasions 11:193–204.

[ece31956-bib-0054] Shea, K. , and P. Chesson . 2002 Community ecology theory as a framework for biological invasions. Trends Ecol. Evol. 17:170–176.

[ece31956-bib-0055] Sousa, W. 1979 Disturbance in marine intertidal boulder fields: the non‐equilibrium maintenance of species diversity. Ecology 60:1225–1239.

[ece31956-bib-0056] Stachowicz, J. J. , H. Fried , R. W. Osman , and R. B. Whitlatch . 2002 Biodiversity, invasion resistance, and marine ecosystem function: reconciling pattern and process. Ecology 83:2575–2590.

[ece31956-bib-0057] Symstad, A. J. 2000 A test of the effects of functional group richness and composition on grassland invasibility. Ecology 81:99–109.

[ece31956-bib-0058] Tamburello, L. , F. Bulleri , D. Balata , and L. Benedetti‐Cecchi . 2014 The role of overgrazing and anthropogenic disturbance in shaping spatial patterns of distribution of an invasive seaweed. J. Appl. Ecol. 51:406–414.

[ece31956-bib-0059] Underwood, A. J. 1991 Beyond BACI: experimental designs for detecting human environmental impacts on temporal variations in natural populations. Aust. J. Mar. Freshw. Res. 42:569–597.

[ece31956-bib-0060] Wardle, D. A. 2001 Experimental demonstration that plant diversity reduces invasibility – evidence of a biological mechanism or a consequence of sampling effect? Oikos 95:161–170.

