# Supplementary material for: Linking disturbance and resistance to invasion via changes in biodiversity: a conceptual model and an experimental test on rocky reefs
Source: Ecol Evol. 2016 Feb 25;6(7):2010–21. doi: 10.1002/ece3.1956 (PMC4767907; doi:10.1002/ece3.1956)
Supplement: Supplementary file 1 — Appendix S1. Description of thallus morphology of the different species of Cystoseira. [file ECE3-6-2010-s001.docx]

**Appendix 1.** Description of thallus morphology of the different species of *Cystoseira*

*Cystoseira barbata* (Stackhouse) C. Agardh (Fig. 1A) is a macroalga with a single axis, attached to the substratum by a small circular disc. The axis is cylindrical (usually up to 50 cm in height) and with a smooth apex. Primary and secondary branches are well-developed, especially in spring.

*Cystoseira crinita* Duby (Fig. 1B) is a caespitose macroalga, with a thallus usually 25-30 cm in height. The thallus is attached to the substratum by a discoid base from which erect axes (usually about 12) are generated. Apices of the axes are with spines. Axes are characterized by the presence of scars, which represent remnants of primary branches. Primary branches have small spines at the base, while secondary and tertiary branches are very thin, normally without spinose appendages.

*Cystoseira compressa* (Esper) Gerloff *&* Nizamuddin (Fig. 1C) is a caespitose macroalga attached to the substratum through a small disc. Axes are usually very short, 1-5 cm, and with smooth apices. In spring and summer primary and secondary branches are longer, usually around 60 cm and, in some urban areas, the thallus can reach more than 2 m in height. In autumn and winter individuals are smaller, usually rosette-shaped (up to 10-12 cm in height), with flattened primary branches.

A)

B)

C)
