# Supplementary material for: Linking disturbance and resistance to invasion via changes in biodiversity: a conceptual model and an experimental test on rocky reefs
Source: Ecol Evol. 2016 Feb 25;6(7):2010–21. doi: 10.1002/ece3.1956 (PMC4767907; doi:10.1002/ece3.1956)
Supplement: Supplementary file 2 — Appendix S2. ANOVAs comparing the cover of the different canopy‐forming macroalgae and species richness among treatments. [file ECE3-6-2010-s002.docx]

**Appendix 2.** Variation in the richness and abundance of canopy-forming macroalgae among experimental treatments

Table S2. Analysis of variance comparing the cover of the different canopy-forming macroalgae and their richness between total canopy removal plots (indicated as TR in the table) and the other treatments, among the other treatments (7 levels) and between control and disturbed plots. ** *P* < 0.01; *** *P* < 0.01

Source of variation *C. barbata* *C. compressa* *C. crinita* Species richness

df MS *F* MS *F* MS *F* MS *F*

TR vs Others^a^ 1 1969.83 2.96 4382.500 7.51** 1376.600 2.86 4.576 10.47**

Treatment = T 6 18.845 24.10*** 3339.767 0.47 3692.720 31.87*** 1.934 6.50***

Disturbance = D 1 0.269 0.34 126.000 0.47 360.071 3.11 0.071 0.24

T x D 6 0.968 1.24 280.074 1.04 90.868 0.78 0.196 0.66

Residual 42 0.782 269.095 115.881 0.298

^a^Tested on the residual (df = 58; MS: *C. barbata* = 666.23; *C. compressa =* 2.336; *C. crinita* = 2.746; species richness = 25.357 )

Fig. S2. Final number of canopy-forming macroalgae in plots exposed to different levels of disturbance intensity, separately for plots that were composed by 1, 2 or 3 species, respectively, at the beginning of the experiment. *n* = 12 for 1- and 2-species assemblages; *n* = 4 for 3-species assemblages.
