# Supplementary material for: Linking disturbance and resistance to invasion via changes in biodiversity: a conceptual model and an experimental test on rocky reefs
Source: Ecol Evol. 2016 Feb 25;6(7):2010–21. doi: 10.1002/ece3.1956 (PMC4767907; doi:10.1002/ece3.1956)
Supplement: Supplementary file 3 — Appendix S3. ANOVA assessing the effects of disturbance, canopy species richness and composition on Caulerpa. [file ECE3-6-2010-s003.docx]

**Appendix 3.** ANOVA assessing the effects of disturbance, canopy species richness and composition on *Caulerpa*

Table S3. Analysis of variance of the effects of the richness (2 levels; 1 vs 2 species) and composition (3 levels for each level of species number) of canopy stands and disturbance (2 levels; controls versus disturbed) on the percentage cover of *Caulerpa*.

Source of variation df MS F

Richness = R 1 315.187 0.10

Composition (R) = C(R) 4 3067.507 9.32***

Disturbance = D 1 406.391 0.32

R x D 1 1340.558 1.06

C(R) x D 4 1263.738 3.84*

Residual 36 328.961

Cochran’s test *P* = 0.201

Transformation None
