# Supplementary material for: Linking disturbance and resistance to invasion via changes in biodiversity: a conceptual model and an experimental test on rocky reefs
Source: Ecol Evol. 2016 Feb 25;6(7):2010–21. doi: 10.1002/ece3.1956 (PMC4767907; doi:10.1002/ece3.1956)
Supplement: Supplementary file 4 — Appendix S4. Relationships between Caulerpa and Cystoseira spp. cover in fall 2010. [file ECE3-6-2010-s004.docx]

**Appendix 4.** Relationships between *Cystoseira* spp. and *C. cylindracea* covers in fall 2010

During fall and winter, thalli of both *C. barbata* and *C. crinita* exhibit a simpler architectural complexity, in part due to the loss of secondary fronds, resulting in a lower canopy cover. Likewise, *C. compressa* undergoes major seasonal changes in morphology; although not necessarily resulting in lower cover, most of the thalli are characterized by a rosette-form (i.e. short stipe, flattened primary branches and short secondary branches) during fall and winter. Assemblages at our study sites (i.e., along the south coast of the Island of St. Juraj) were sampled in October 2010, in order to assess how the effects of the different canopy-forming species (*C. barbata*, *C. compressa* and *C. crinita*) on *C. cylindracea*, experimentally evaluated when their canopy development is maximum, compare to those they have on the invader when the architecture of their thalli is lower. The covers of *C. cylindracea* and that of the canopy-forming species were sampled in 48, 50 x 50 cm quadrats, randomly scattered across the study site, by means of the technique described in the main text. The relationship between the cover of *C. cylindracea* and that of the canopy-formers was analyzed by means of Spearman’s rank correlation. The cover of *C. cylindracea* was negatively correlated to that of *C. compressa* (ρ = -0.41; *P* = 0.004; Fig. 1A), it was not correlated to that of *C. barbata* (ρ = -0.20; *P* = 0.177; Fig. 1B) and it was positively correlated to that of *C. crinita* (ρ = 0.35; *P* = 0.016; Fig. 1C). Although these data cannot establish a cause-effect relationship, they suggest that the direction of the effects of the different species of *Cystoseira* is consistent across times of the year at which thalli differ in morphology.
